# Evolving Hybrid Partial Genetic Algorithm Classification Model for Cost-effective Frailty Screening: Investigative Study

**DOI:** 10.2196/38464

**Published:** 2022-10-07

**Authors:** John Oates, Niusha Shafiabady, Rachel Ambagtsheer, Justin Beilby, Chris Seiboth, Elsa Dent

**Affiliations:** 1 Torrens University Ultimo Australia; 2 College of Engineering, Information Technology and Environment Charles Darwin University Haymarket, NSW Australia; 3 Torrens University Adelaide Australia; 4 Lutheran Services Milton Australia

**Keywords:** machine learning, frailty screening, partial genetic algorithms, SVM, KNN, decision trees, frailty, algorithm, cost, model, index, database, ai, ageing, adults, older people, screening, tool

## Abstract

**Background:**

A commonly used method for measuring frailty is the accumulation of deficits expressed as a frailty index (FI). FIs can be readily adapted to many databases, as the parameters to use are not prescribed but rather reflect a subset of extracted features (variables). Unfortunately, the structure of many databases does not permit the direct extraction of a suitable subset, requiring additional effort to determine and verify the value of features for each record and thus significantly increasing cost.

**Objective:**

Our objective is to describe how an artificial intelligence (AI) optimization technique called partial genetic algorithms can be used to refine the subset of features used to calculate an FI and favor features that have the least cost of acquisition.

**Methods:**

This is a secondary analysis of a residential care database compiled from 10 facilities in Queensland, Australia. The database is comprised of routinely collected administrative data and unstructured patient notes for 592 residents aged 75 years and over. The primary study derived an electronic frailty index (eFI) calculated from 36 suitable features. We then structurally modified a genetic algorithm to find an optimal predictor of the calculated eFI (0.21 threshold) from 2 sets of features. Partial genetic algorithms were used to optimize 4 underlying classification models: logistic regression, decision trees, random forest, and support vector machines.

**Results:**

Among the underlying models, logistic regression was found to produce the best models in almost all scenarios and feature set sizes. The best models were built using all the low-cost features and as few as 10 high-cost features, and they performed well enough (sensitivity 89%, specificity 87%) to be considered candidates for a low-cost frailty screening test.

**Conclusions:**

In this study, a systematic approach for selecting an optimal set of features with a low cost of acquisition and performance comparable to the eFI for detecting frailty was demonstrated on an aged care database. Partial genetic algorithms have proven useful in offering a trade-off between cost and accuracy to systematically identify frailty.

## Introduction

Genetic algorithms (GA) are a general-purpose computational optimization method inspired by the evolution mechanism in nature. They are one of the most popular metaheuristic search algorithms and have been used for variety of applications, including synthetic data generation, feature selection, and to solve complex equations [1]. In this study, genetics algorithms have been applied to identify features that offer a suitable trade-off between cost and accuracy.

Within the context of global population aging, the number of older people who will live a significant proportion of their lives with frailty is growing rapidly [2]. Frailty is problematic for older people and the societies in which they live due to the elevated risks associated with the syndrome, including terms poor health outcomes [3] and additional use of health and aged care services [4-7], leading to inflated health care costs [8-10]. However, emerging research suggests that frailty is a highly dynamic [11,12] and potentially modifiable state with appropriate intervention [13,14]. Screening for early detection is proposed to increase the likelihood that the worst impacts of frailty can be lessened [4,15,16].

There are 2 main approaches to identifying frailty: the frailty phenotype (FP) and the frailty index (FI) [17]. However, these established approaches have known drawbacks, requiring significant time investment, face-to-face interaction, and specific data items to be collected [18]. Recently, an electronic frailty index (eFI) was proposed [19] that has the potential to achieve greater efficiencies over face-to-face models when applied to administrative data sets, but the need to ensure a minimum set of items adhering to prespecified criteria remains a barrier to implementation. For example, previous research has shown that although it is possible to calculate and construct an eFI based on an aged care administrative data set, a significant proportion of the items require manual calculation to ensure accuracy and improve quality [20]. Clearly, it would be preferable to identify automated techniques capable of delivering comparable accuracy and quality but with greater efficiency. Consequently, this study aimed to apply a sophisticated genetic algorithm technique to identify an optimal predictor of the calculated eFI.

## Methods

### Study Design, Participants, and Setting

This retrospective study utilized a data set previously compiled [21] from the administrative database of 10 residential aged care facilities located in Queensland, Australia. Participants were included in the study if they were aged 75 years or older and had completed an Aged Care Funding Instrument (ACFI) assessment within the previous 3 years.

### Ethical Considerations

A waiver of consent for the initial study was obtained from the Human Research Ethics Committee of Torrens University Australia (application H11/19), which declared the study exempt under National Statement 5.1.22 (secondary use of deidentified administrative data) due to the pragmatic nature of the study. Because this is a secondary study of the same data, the approval extends to this study. Moreover, this study adheres to the Australian National Statement on Ethical Conduct in Human Research.

### Frailty Outcome Measure

An eFI was previously calculated for this data [21] based on a formulation originally specified by Clegg et al [22]. Care was taken to ensure the included deficits adhered to the criteria recommended by Searle and colleagues [23], which resulted in 32 of the 35 deficits being extracted from unstructured patient notes and only 3 being derived from the ACFI data. The binary frailty classification was derived using a threshold of 0.21 (ie, frailty defined as >0.21) [24].

### Screening Test Construction

Genetic algorithms are an optimization technique [1] applied in machine learning to filter a set of features that are used to construct a classification model. During training, a classification algorithm is tuned on a training set, and the success of attaining a generalized predictive algorithm is then verified by measuring the classification errors in the test set.

Genetic algorithms leverage the observation that classification models often perform better when they are trained on a subset of the available features. Which subset of features to use, however, is not obvious. Genetic algorithms start with a population of randomly generated subsets of features, or chromosomes, that are all independently used to generate classification models. The chromosomes from the population that generated the best performing models are allowed to combine, or breed, to form a new generation of the population, while the worst performing ones are removed completely. The process continues until either a predefined number of generations have been trained or the performance of the models has plateaued. Once training is complete, the best-performing model is deployed using only the naturally selected subset of the available features.

While genetic algorithms are good at selecting an optimal subset of features, they select the features based on maximizing the classification accuracy of a generated model. The cost of acquiring the various features is not factored into the choice of features, even if the performance of less expensive features is close to that of their more expensive counterparts. In this study, the cost of a feature is the combination of the effort, monetary cost, and patient risk involved in capturing the values. We want to minimize the number of expensive features chosen to form the model but allow as many low-cost features to be used as is necessary to gain acceptable performance of the model.

**Figure 1:**
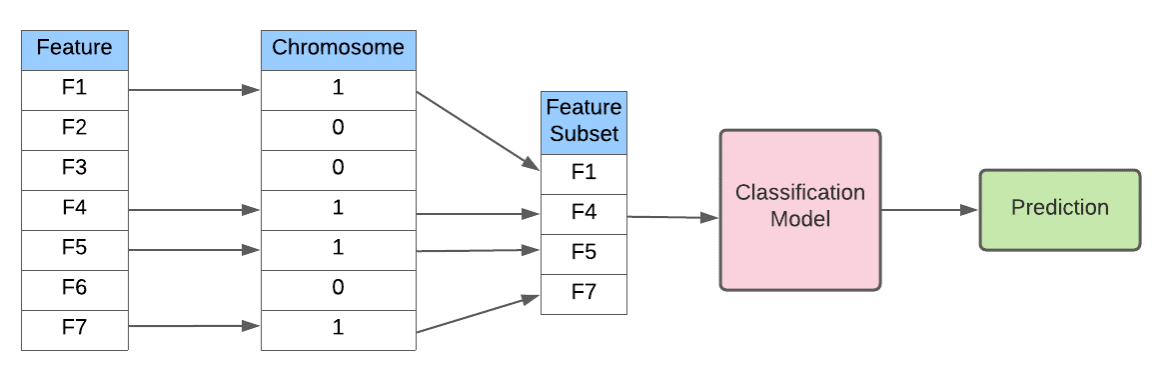
Genetic algorithm configuration for training a single member of the population.

**Figure 2:**
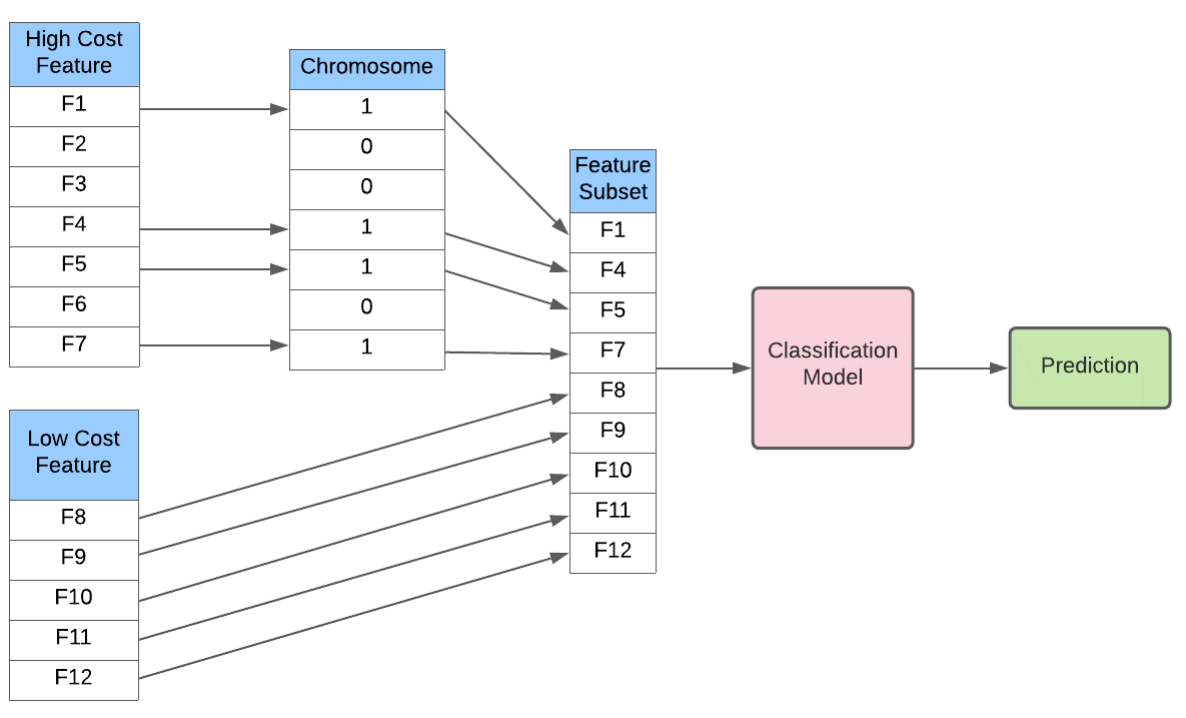
Partial genetic algorithm configuration for training a single member of the population.

To achieve the inclusion of low-cost features in the classification model, the standard genetic algorithm training configuration illustrated in Figure 1 is modified as illustrated in Figure 2.

This modification is performed every time a model is trained for every member of the population trialed by the genetic algorithm. When the genetic algorithm trains a model, it passes a subset of the available training records to the classification model’s training algorithm. The low-cost feature values for each record need to be added to the selected training records before commencing the training. The genetic algorithm trains the classification model for each chromosome multiple time with different subsets of the training records and determining the performance of each model using records not used in training that instance. As with the training records, the low-cost features need to be added to the records used to determine a model’s performance. The performance of the chromosome is calculated as the average performance of all the models built from different subsets of the training records. This process is called n-fold cross validation, where n is the number of models built. In this study, 3-fold cross validation was used because it ensured a good balance between performance and the time it took to build the models.

Four types of classification models were optimized using partial genetic algorithms: logistic regression, support vector machines, random forest, and decision trees. These algorithms are popular choices for classification because they have proven successful in generating generalized models for a wide range of applications [20]. Logistic regression is a statistical modeling technique whereby a linear combination of the input features is found during training, which models the logarithm of the odds that a binary outcome is in the true state. A support vector machine (SVM) aims to learn a multidimensional hyperplane that separates the set of records given to it for training. Predictions are made by placing the candidate record in the same multidimensional classification space and determining which side of the hyperplane it maps to. SVM was developed in the 1990s and has since enjoyed success in many real-world applications, including pattern recognition [25], text classification [26], and bioinformatics. Decision trees employ a divide and conquer strategy. A tree is formed of nodes, and each node performs a comparison of a single input feature and a threshold if the variable is continuous or a state if the feature is discrete. The outcome of the comparison determines the choice of the next node, which either performs a new comparison or terminates the tree with a given classification. During training, the set of training records are used to find comparisons at each node that gain the most information by reducing entropy in the outcomes by the greatest amount. Subsequent training predictions are made by feeding records into the root node and determining the classification of the terminating node where the record exits the tree. Random forest is a meta form of decision trees, where the output is determined by a vote between many trees. The trees are built using different methods to ensure they are not replicas of each other.

The software was written in Python and the models were built using the sklearn module (version 1.0.2) and the genetic_selection module from sklearn-genetic (version 0.5.1).

## Results

### Model Generation

Of the 69 features considered, 34 were extracted directly from the ACFI assessment and 35 were the values used to calculate the eFI. Two of the ACFI features, Psychogeriatric Assessment Scales (PAS) score and Cornell Scale, were excluded as they had a high percentage of missing values (PAS score 36%, Cornell Scale 42%). The remaining 32 ACFI assessment features had no missing values and were categorized as low cost of acquisition features. Of the 35 features used to calculate the eFI, 32 were extracted by an automated search for key words in the unstructured patient notes, followed by manual inspection and verification by a clinician. These were categorized as having a high cost of acquisition. The remaining 3 features used to calculate the eFI were direct combinations of ACFI features. As the calculation of these features could be fully automated, they were included with the low-cost features. A total of 4 sets of low-cost features were considered: (1) ACFI features + the low-cost eFI features; (2) the low-cost eFI features; (3) no low-cost features; and (4) a set of features chosen from the low-cost features using genetic algorithms. A different set was found for each of the classification algorithms.

**Figure 3:**
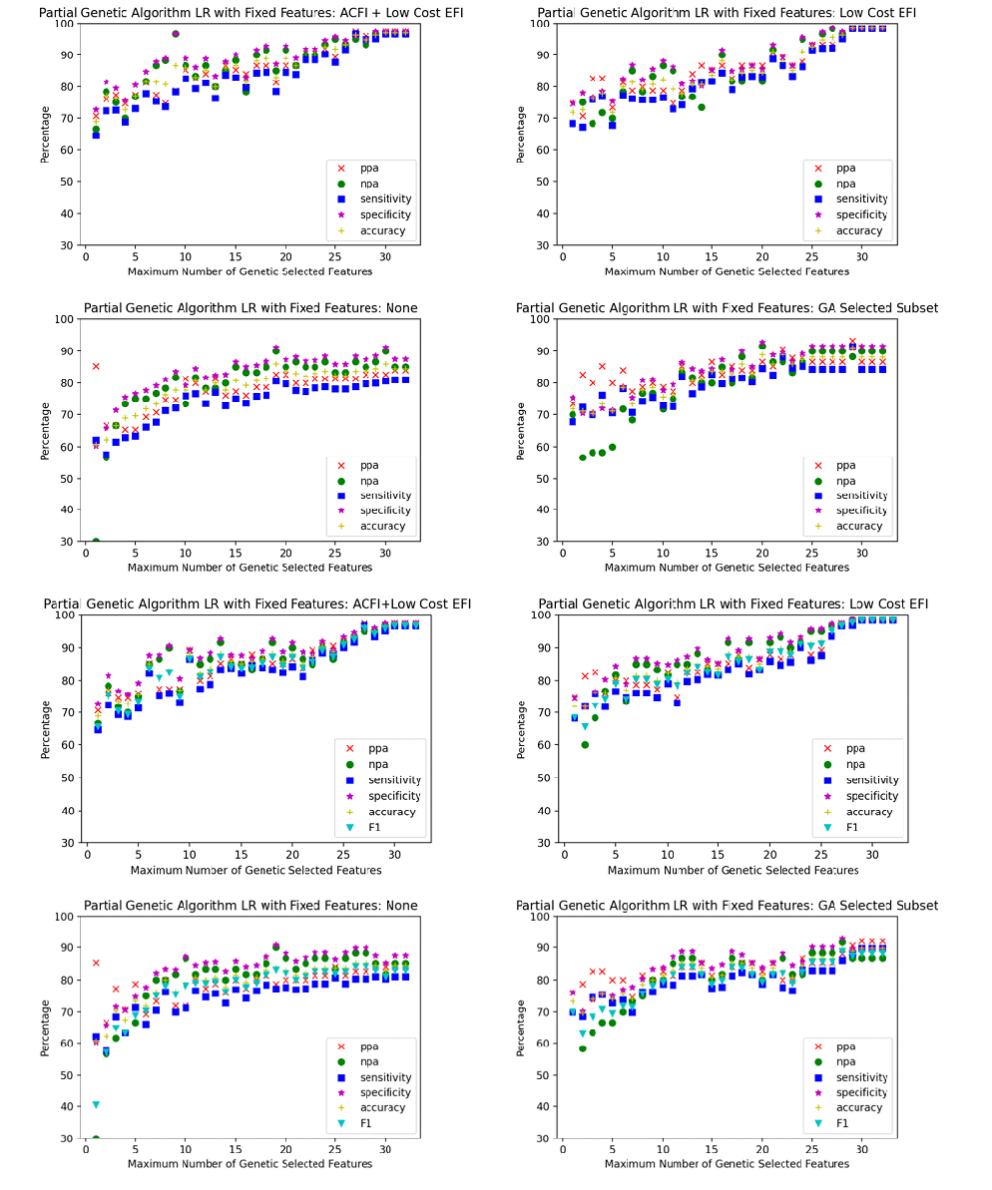
Logistic regression optimized with a partial genetic algorithm. ACFI: Aged Care Funding Instrument; EFI: electronic frailty index; GA: Genetic algorithm; LR: logistic regression; npa: negative percent agreement; ppa: positive percent agreement.

**Figure 4:**
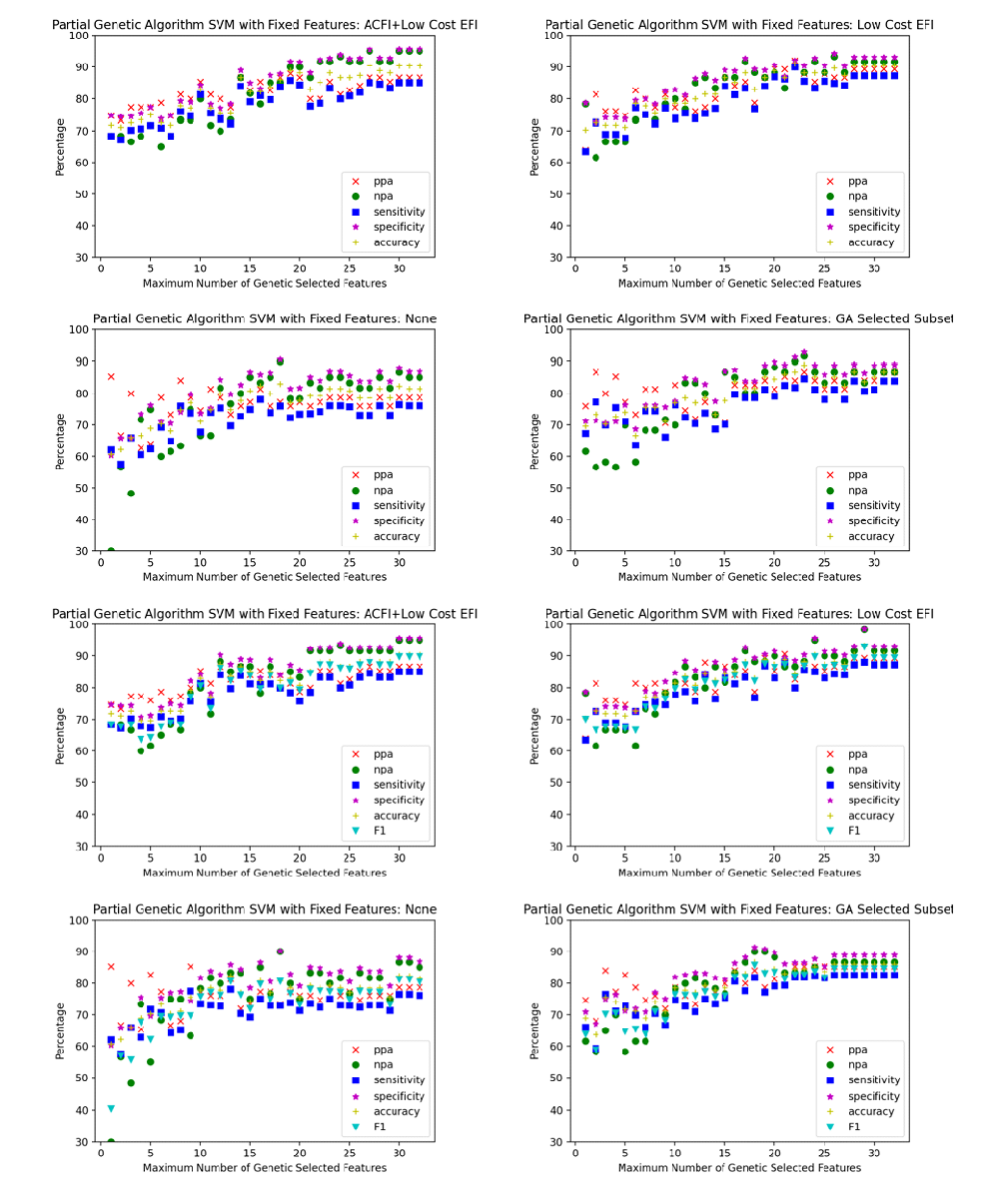
Support vector machine optimized with a partial genetic algorithm. ACFI: Aged Care Funding Instrument; EFI: electronic frailty index; GA: Genetic algorithm; npa: negative percent agreement; ppa: positive percent agreement; SVM: support vector machine.

**Figure 5:**
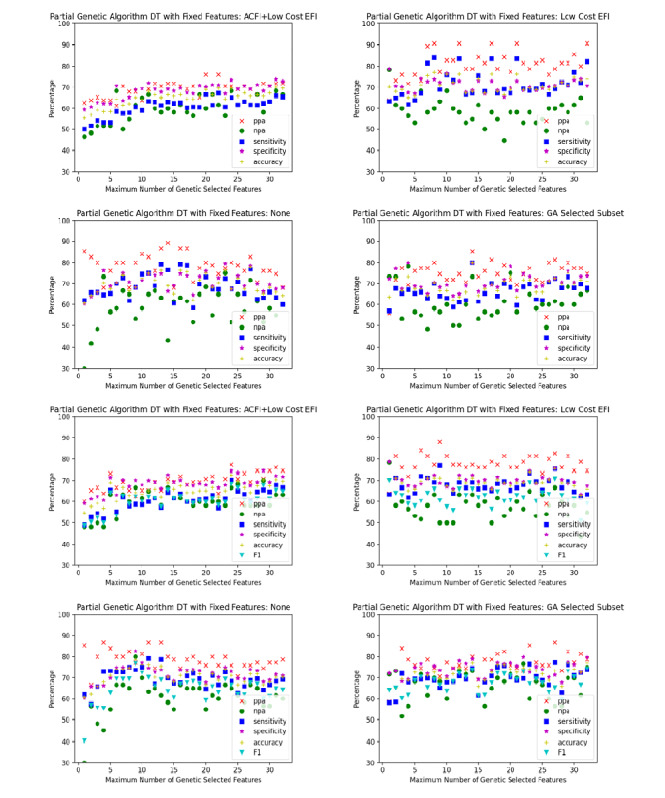
Decision tree optimized with a partial genetic algorithm. ACFI: Aged Care Funding Instrument; DT: decision tree; EFI: electronic frailty index; GA: Genetic algorithm; npa: negative percent agreement; ppa: positive percent agreement.

**Figure 6:**
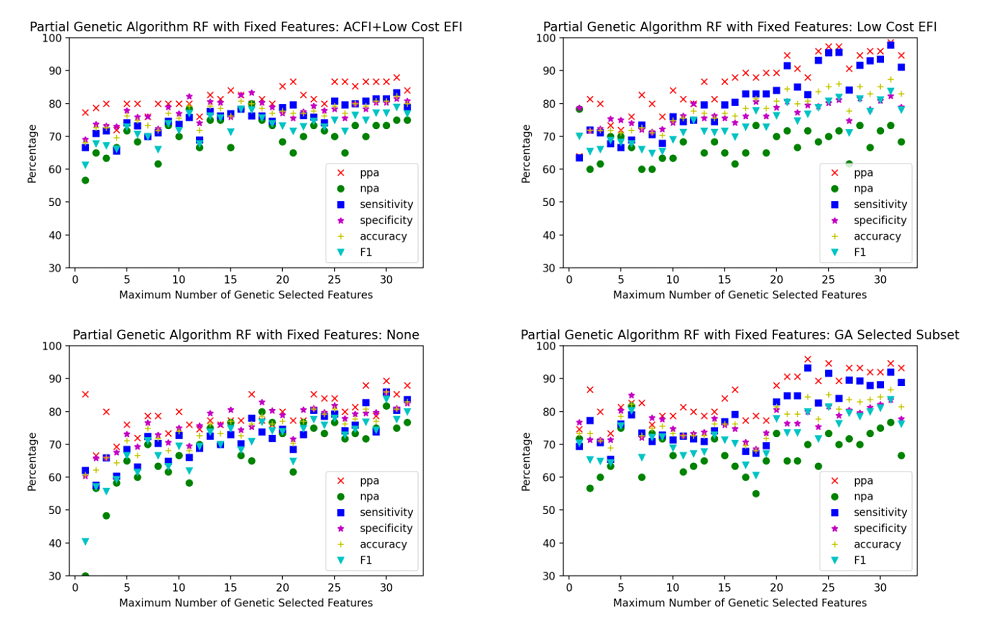
Random forest optimized with a partial genetic algorithm. ACFI: Aged Care Funding Instrument; EFI: electronic frailty index; GA: Genetic algorithm; npa: negative percent agreement; ppa: positive percent agreement; RF: random forest.

Sixteen scenarios were trialed, comprising each of the aforementioned 4 sets of low-cost features for each of the 4 classification algorithms. For each scenario, the partial genetic algorithm was used to optimize the classification algorithm with different limits placed on the number of high-cost features. The limits were varied sequentially from 1 to 32, which was the number of candidate high-cost features. The performance of each of the 32 algorithms generated for each scenario were plotted on a single graph. The graphs for each scenario are plotted in Figures 3-6.

When comparing the graphs for each classification model, logistic regression outperformed decision trees in every scenario and SVM and random forest in almost all scenarios. Tables 1-3 demonstrate the numeric comparison of the 16 scenarios when 5, 10, and 15 of the high cost of acquisition features were used.

The option of “No low-cost” features was provided to determine how much predictive value the low-cost features were adding to the classification. As expected, this option performed the worst for all the classification algorithms, confirming that the low-cost features were adding value. Next, models were built using only the 3 low-cost eFI features as fixed features. This improved the accuracy of the logistic regression algorithm to 97% when almost all the eFI features were included (Table 4). Although this is a good outcome, a model built using so many of the high-cost features was not the goal of this study.

A genetic algorithm works by selecting an optimal subset of all the features made available to it. This characteristic was the motivation behind building a version of the models in 2 stages. In the first stage, a standard, nonpartial, genetic algorithm was used on the low-cost features to find an optimal combination. These models performed so poorly (Table 5) that they could not be used without further improvement. The combination of features used to generate these models (Multimedia Appendices 1-3) was then employed as the fixed features in the partial genetic algorithm during the second stage. The models in the second stage performed surprisingly poorly, showing no difference from the models built without any low-cost features, regardless of the classification model used.

Using all the low-cost features in a partial genetic algorithm yielded the best overall results and matched the 97% accuracy achieved by the models that used the low-cost eFI features when the model was able to select most of the high-cost eFI features. At 10 features, however, the extra low-cost features allowed the algorithm to increase its sensitivity from 82.7% to 89.3% and specificity from 81.7% to 86.7%.

**Table 1:** Performance of the 12 scenarios with 5 high-cost features.

Features		Sensitivity	Specificity	PPA^a^	NPA^b^	Accuracy	F1^c^
**ACFI^d^ + low-cost eFI^e^**							
	Logistic regression	76	75	71.4	79.2	75.6	73.2
	Support vector machine	76	61.7	67.3	71.3	69.6	64.3
	Decision tree	73.3	63.3	65.5	71.4	68.8	64.4
	Random forest	80	71.7	74.1	77.9	76.3	72.9
**Low-cost eFI**							
	Logistic regression	80	81.7	76.6	84.5	80.7	79
	Support vector machine	74.7	66.7	67.8	73.7	71.1	67.2
	Decision tree	76	53.3	64.0	67.1	65.9	58.2
	Random forest	72	70	66.7	75	71.1	68.3
**No low-cost features**							
	Logistic regression	78.7	66.7	71.4	74.7	73.3	69
	Support vector machine	82.6	55	71.2	69.7	70.4	62.2
	Decision tree	84	55	73.3	70	71.1	62.9
	Random forest	76	65	68.4	73.1	71.1	66.7
**Genetically selected low-cost features**							
	Logistic regression	80	66.7	72.7	75	74.1	69.6
	Support vector machine	82.7	58.3	72.9	71.3	71.9	64.8
	Decision tree	76	68.3	69.5	75	77.2	68.9
	Random forest	81.3	75	76.3	80.3	78.5	75.6

^a^PPA: positive percent agreement.

^b^NPA: negative percent agreement.

^c^F1: F-score.

^d^ACFI: Aged Care Funding Instrument.

^e^eFI: electronic frailty index.

**Table 2:** Performance of the 12 scenarios with 10 high-cost features.

Features			Sensitivity		Specificity		PPA^a^	NPA^b^		Accuracy		F1^c^	
**ACFI^d^ + low-cost eFI^e^**													
	Logistic regression	89.3		86.7		86.7		89.3	88.1		86.7		
	Support vector machine	85.3		80.0		81.4		84.2	83		80.7		
	Decision tree	65.3		61.7		58.7		68.1	63.7		60.2		
	Random forest	80		70		73.9		76.9	75.6		71.8		
**Low-cost eFI**													
	Logistic regression	82.7		81.7		79		84.9	82.2		80.3		
	Support vector machine	81.3		81.7		77.8		84.7	81.5		79.7		
	Decision tree	81.3		50		68.2		67	67.4		57.7		
	Random forest	84		63.3		76		74.1	74.8		69.1		
**No low-cost features**													
	Logistic regression	72		86.7		71.2		87.1	78.5		78.2		
	Support vector machine	77.3		78.3		73.4		81.7	77.8		75.8		
	Decision tree	81.3		70		75		77.2	76.3		72.4		
	Random forest	80		66.7		72.7		75	74.1		69.6		
**Genetically selected low-cost features**													
	Logistic regression	82.6		80		78.6		83.8	81.5		79.3		
	Support vector machine	78.7		78.3		74.6		81.9	78.5		76.4		
	Decision tree	77.3		60		67.9		70.7	69.6		63.7		
	Random forest	78.7		66.7		71.4		74.7	73.3		69		

^a^PPA: positive percent agreement.

^b^NPA: negative percent agreement.

^c^F1: F-score.

^d^ACFI: Aged Care Funding Instrument.

^e^eFI: electronic frailty index.

**Table 3:** Performance of the 12 scenarios with 15 high-cost features.

Features			Sensitivity		Specificity		PPA^a^		NPA^b^		Accuracy		F1^c^
**ACFI^d^ + low-cost eFI^e^**													
	Logistic regression	85.3		85.0		82.3		87.7		85.2		83.6	
	Support vector machine	84.0		86.7		81.3		88.7		85.1		83.9	
	Decision tree	69.3		61.7		61.7		69.3		65.9		61.7	
	Random forest	84.0		66.7		76.9		75.9		76.3		71.4	
**Low-cost eFI**													
	Logistic regression	85.3		81.7		81.7		85.3		83.7		81.7	
	Support vector machine	86.7		81.7		83.1		85.5		84.4		82.4	
	Decision tree	76.0		58.3		66.0		69.5		68.1		61.9	
	Random forest	86.7		65.0		79.6		75.6		77.0		71.6	
**No low-cost features**													
	Logistic regression	80.0		83.3		76.9		85.7		81.5		80.0	
	Support vector machine	73.3		75.0		69.2		78.6		74.1		72.0	
	Decision tree	78.7		55.0		67.3		68.6		68.1		60.6	
	Random forest	77.3		76.6		73.0		80.6		77.0		74.8	
**Genetically selected low-cost features**													
	Logistic regression	81.3		80.0		77.4		83.5		80.7		78.7	
	Support vector machine	80.0		76.7		75.4		81.1		78.5		76.0	
	Decision tree	69.3		61.7		61.7		69.3		65.9		61.7	
	Random forest	84.0		66.7		76.9		75.9		76.3		71.4	

^a^PPA: positive percent agreement.

^b^NPA: negative percent agreement.

^c^F1: F-score.

^d^ACFI: Aged Care Funding Instrument.

^e^eFI: electronic frailty index.

**Table 4:** Performance of models based on all features.

Algorithm	Sensitivity	Specificity	PPA^a^	NPA^b^	Accuracy	F1^c^
LR^d^	97.3	96.7	96.7	97.3	97.0	96.7
SVM^e^	86.7	95.0	85.1	95.6	90.4	89.8
DT^f^	76.0	63.3	67.9	72.1	70.4	65.5
RF^g^	88.0	75.0	83.3	81.5	82.2	78.9

^a^PPA: positive percent agreement.

^b^NPA: negative percent agreement.

^c^F1: F-score.

^d^LR: logistic regression.

^e^SVM: support vector machine.

^f^DT: decision tree.

^g^RF: random forest.

**Table 5:** Performance of models based only on low-cost features.

Algorithm	Sensitivity	Specificity	PPA^a^	NPA^b^	Accuracy	F1^c^
LR^d^	77.3	63.3	69.1	72.5	71.1	66.1
SVM^e^	77.3	58.3	67.3	69.9	68.9	62.5
DT^f^	61.3	70.0	59.2	71.9	65.2	64.1
RF^g^	77.3	58.3	67.3	69.9	68.9	62.5

^a^PPA: positive percent agreement.

^b^NPA: negative percent agreement.

^c^F1: F-score.

^d^LR: logistic regression.

^e^SVM: support vector machine.

^f^DT: decision tree.

^g^RF: random forest.

## Discussion

### Principal Findings

With AI techniques, cost-effective screening tests for frailty are possible for aged care databases that contain an ACFI assessment and unstructured patient notes. This study has shown that the ACFI assessment alone does not provide sufficient information to determine if a patient is frail. However, when ACFI data are augmented by as few as 10 additional features, an AI model can be derived that performs well enough to be used as a screening test. What this means in clinical practice is that older people with frailty can be rapidly and accurately identified in residential care using our novel AI-derived model for frailty. A rapid identification of frailty is crucial to optimally manage the condition [27]. Indeed, the recent Australian Royal Commission to Aged Care highlighted the importance of early identification of aged care residents with frailty, who require additional support [28].

The value of any AI-derived model for frailty screening can be judged by the amount it reduces the cost of acquisition of the features required to determine the value of the deficits used to construct a frailty index. Features that are routinely collected and stored in a database in a format that can be directly fed into a classification model have a low cost of acquisition. Unfortunately, as shown in this study (Table 5) and others [20], such models lack both the sensitivity and specificity to be useful screening tests. At the other extreme, models that include all the deficit features used to calculate the eFI perform extremely well [20] (Table 4), but the value of such models is marginal.

To be useful for a screening test, a model must be acceptably accurate and significantly reduce the cost of acquisition of the features required to implement a frailty index. If a model cannot be developed with acceptable accuracy without including at least some high-cost features, it is desirable to determine the optimal minimum set of high-cost features required to achieve an acceptable performance. Genetic algorithms perform well at determining the optimal subset of features required to maximize the performance of a model. Furthermore, their choice of a subset can be limited to any number of features, up to and including all the available features. This allows the trade-off between the number of features and the performance of the derived models to be determined.

This study found that if a genetic algorithm was permitted to choose any number of features from all the available features, regardless of their cost, it most frequently chose subsets that only included high-cost features. This motivated the development of the previously mentioned partial genetic algorithm, which forced the algorithm to include low-cost features as well. However, this raises the question of whether the low-cost features add any value at all. To answer this question, the results include both a fixed set that had no low-cost features and a set that included only the low-cost features used to calculate the eFI. Considering logistic regression models with 10 high-cost features, including all the low-cost features, yielded an improvement of 17% in sensitivity (89% versus 72%). This combination did not compromise specificity, which remained stable (87%) and is comparable to the scenario with no low-cost features. This improvement is significant and possibly represents the difference between a clinically useful screening test and one that is inadequate. Even if the comparison is made between models built on all the low-cost features and those that include only low-cost features used in the eFI calculation, there is a 6% improvement in sensitivity (89% versus 83%) and 5% in specificity (87% versus 82%).

Although the partial genetic algorithm–built models with 10 high-cost features use less than a third of all the high-cost features, they still require those 10 features to be extracted by screening patient notes. Recent advances in natural language processing (NLP) show promise for automating this extraction process. It is plausible that NLP could extract all the features required to calculate the eFI, but this would require a much larger data set than the one used in this study. In the meantime, the cost of acquisition of at least 10 features from every patient record remains the cost of implementing a screening test on any database similar to ours that contains an ACFI assessment and unstructured patient notes.

Partial genetic algorithms can be used to derive classification models from any database where the cost of acquisition of some parameters is higher than it is for others. Although they have been demonstrated in this study on an aged care database to predict frailty, they could be used in any domain. They are well suited to permit AI models to be trained to implement screening tests in domains where costs are important and there is a difference in the cost of acquisition of candidate features.

### Limitations

Because this study reuses the data from a previous study [20], it shares the limitations associated with the data from the first study. In particular, the data were sourced from a single aged care provider, and the data set was relatively small. This study further filtered patients based on the availability of an ACFI assessment. It is plausible that these criteria gave a skewed representation of the population that a screening test would be applied to, resulting in different model performance. The ability to reproduce AI results continues to be controversial [29,30] within medicine, so further studies should aim to reproduce these results with different data sets. A further limitation is the changing model of aged care in Australia, with a new model set to replace ACFI in the next 2 years.

### Conclusion

The value of screening tests lies in their cost-effective application. The main cost of applying a model-based screening test lies in acquiring the measures fed into the model. To derive useful screening tests using AI techniques, algorithms must be employed that favor the use of cheaper features over those that require more effort or patient risk to acquire. What all aged care providers and their clinical advisers need is a screening tool that will allow the efficient planning of evidence-based interventions to older frail people who will best benefit from them. At a time where the aged care sector and all providers are being asked by governments and national quality agencies to focus on this vulnerable group, it is crucial that we employ an efficient screening tool.

This paper has shown how partial genetic algorithms can be used to determine an optimal subset of high-cost features to use with cheap features to derive AI models to classify frailty, both in terms of which parameters to use and how many to use. This technique can be applied to any database. It does not guarantee that an adequate model will be found from any database, but it does give a good indication of whether there is sufficient information in the data to derive a model.

Partial genetic algorithms were demonstrated in this paper to derive a cost-effective screening test for frailty, but the method can be applied to any screening tests where there is a disparity in the cost of measuring the required features. The outcome of this study will aid health care providers in screening for frailty with better accuracy through the proposed cost-effective method, which strikes a good balance between accuracy and cost.

## Appendix

Multimedia Appendix 1 Full list of features.

Multimedia Appendix 2 Selected features.

Multimedia Appendix 3 Low-cost features selected for models built with GA-selected subset.

## Abbreviations

ACFI: Aged Care Funding Instrument
AI: artificial intelligence
eFI: electronic frailty index
FI: frailty index
FP: frailty phenotype
GA: genetic algorithm
NLP: natural language processing
PAS: Psychogeriatric Assessment Scales
SVM: support vector machine
