# Supplementary material for: Evolving Hybrid Partial Genetic Algorithm Classification Model for Cost-effective Frailty Screening: Investigative Study
Source: JMIR Aging. 2022 Oct 7;5(4):e38464. doi: 10.2196/38464 (PMC9587492; doi:10.2196/38464)
Supplement: Multimedia Appendix 1 [file aging_v5i4e38464_app1.docx]

Multimedia Appendix 1: Full List of Features

| ACFI Features: | Low Cost EFi Features: | High Cost EFi Features: |
| --- | --- | --- |
| Age | Eating Assistance | Anorexia |
| Gender | Mobility | Arthritis |
| Activities of Daily Living | Req Care | AF |
| Behaviour Domain |  | Anxiety |
| Complex Health Care Domain |  | CKD |
| Nutrition |  | COPD |
| Mobility result |  | CVA |
| Personal Hygiene |  | Depression |
| Toileting |  | Diabetes |
| Continence |  | Dizziness |
| Cognitive Skills |  | Dysphagia |
| Wandering |  | Dyspnea |
| Medication |  | Falls |
| Complex Health Care |  | Feet |
| Verbal Behaviour |  | Fracture |
| Physical Behaviour |  | Hearing |
| Depression |  | Heart Failure |
| Eating |  | Heart Valve Disease |
| Readiness To Eat |  | Hypertension |
| Transfers |  | Hypotension |
| Locomotion |  | IHD |
| Dressing And Undressing |  | Incontinence |
| Washing And Drying |  | Memory Cognitive |
| Grooming |  | Osteoporosis |
| Use Of Toilet |  | Pain |
| Toilet Completion |  | Parkinsonism |
| Urinary Continence Checklist |  | Peptic |
| Faecal Continence Checklist |  | PVD |
| Checklist |  | Skin Ulcer |
| Verbal Behaviour Checklist |  | Sleep |
| Physical Behaviour Checklist |  | Thyroid |
| Wandering Checklist |  | Vision |
