# Supplementary material for: Evolving Hybrid Partial Genetic Algorithm Classification Model for Cost-effective Frailty Screening: Investigative Study
Source: JMIR Aging. 2022 Oct 7;5(4):e38464. doi: 10.2196/38464 (PMC9587492; doi:10.2196/38464)
Supplement: Multimedia Appendix 2 [file aging_v5i4e38464_app2.docx]

Multimedia Appendix 2: Selected Features

Linear Regression

|  | Algorithm | ACFI + Low Cost EFI | | | Low Cost EFI | | | None | | | GA Selected Subset | | | Usage |
| --- | --- | --- | --- | --- | --- | --- | --- | --- | --- | --- | --- | --- | --- | --- |
| 1 | Number of Features | 5 | 10 | 15 | 5 | 10 | 15 | 5 | 10 | 15 | 5 | 10 | 15 |  |
| 2 | Diabetes | X | X | X | X | X | X | X | X | X | X |  | X | 11 |
| 3 | Depression | X | X | X |  | X | X | X | X | X |  | X | X | 10 |
| 4 | Ihd | X | X |  | X | X | X |  | X |  | X | X | X | 9 |
| 5 | Hypertension | X | X | X | X | X | X |  | X |  | X |  | X | 9 |
| 6 | Af |  | X | X | X | X | X |  |  | X |  | X | X | 8 |
| 7 | Osteoporosis | X | X | X |  |  | X |  | X | X |  |  | X | 7 |
| 8 | Cva |  |  | X |  |  | X | X | X | X |  | X | X | 7 |
| 9 | Incontinence |  |  | X |  |  | X | X | X | X |  | X | X | 7 |
| 10 | Arthritis |  |  | X |  |  | X |  | X | X |  | X | X | 6 |
| 11 | Copd |  |  | X |  | X | X |  |  | X |  | X | X | 6 |
| 12 | Memory Cognitive |  |  |  |  | X | X | X |  | X |  | X | X | 6 |
| 13 | Falls |  | X | X |  | X | X |  |  |  |  | X |  | 5 |
| 14 | Anxiety |  |  | X |  | X | X |  |  |  |  |  | X | 4 |
| 15 | Feet |  | X | X |  |  |  |  | X |  |  | X |  | 4 |
| 16 | Anorexia |  | X | X |  |  |  |  |  |  | X |  |  | 3 |
| 17 | Vision |  |  |  |  | X | X |  |  | X |  |  |  | 3 |
| 18 | Hearing |  | X | X |  |  |  |  |  |  | X |  |  | 3 |
| 19 | Fracture |  |  |  |  |  |  |  | X | X |  |  | X | 3 |
| 20 | Dysphagia |  |  |  |  |  |  |  |  | X |  |  | X | 2 |
| 21 | Dizziness |  |  |  |  |  |  |  |  | X |  |  |  | 1 |
| 22 | Pain |  |  |  |  |  | X |  |  |  |  |  |  | 1 |
| 23 | Parkinsonism |  |  |  |  |  |  |  |  | X |  |  |  | 1 |
| 24 | Pvd |  |  | X |  |  |  |  |  |  |  |  |  | 1 |
| 25 | Sleep |  |  |  |  |  |  |  |  | X |  |  |  | 1 |
| 26 | Thyroid |  |  |  | X |  |  |  |  |  |  |  |  | 1 |
| 27 | Heart Failure |  |  |  |  |  |  |  |  |  |  |  | X | 1 |
| 28 | Heart Valve Disease |  |  |  |  |  |  |  |  |  |  |  |  | 0 |
| 29 | Hypotension |  |  |  |  |  |  |  |  |  |  |  |  | 0 |
| 30 | Dyspnea |  |  |  |  |  |  |  |  |  |  |  |  | 0 |
| 31 | Ckd |  |  |  |  |  |  |  |  |  |  |  |  | 0 |
| 32 | Peptic |  |  |  |  |  |  |  |  |  |  |  |  | 0 |
| 33 | Skin Ulcer |  |  |  |  |  |  |  |  |  |  |  |  | 0 |

Support Vector Machine

|  | Algorithm | ACFI+Low Cost EFI | | | Low Cost EFI | | | None | | | GA Selected Subset | | | Usage |
| --- | --- | --- | --- | --- | --- | --- | --- | --- | --- | --- | --- | --- | --- | --- |
| 1 | Number of Features | 5 | 10 | 15 | 5 | 10 | 15 | 5 | 10 | 15 | 5 | 10 | 15 |  |
| 2 | Depression |  | X | X |  | X | X | X | X | X | X | X | X | 10 |
| 3 | Hearing | X | X | X |  | X | X |  | X | X | X |  |  | 8 |
| 4 | Copd |  |  | X |  | X | X | X | X | X | X | X |  | 8 |
| 5 | Cva |  | X | X |  | X | X |  | X | X |  | X | X | 8 |
| 6 | Diabetes | X | X | X | X |  | X |  | X | X |  |  | X | 8 |
| 7 | Ihd |  | X | X |  | X | X |  | X | X |  | X | X | 8 |
| 8 | Osteoporosis | X | X | X | X |  | X |  |  |  |  | X | X | 7 |
| 9 | Memory Cognitive |  | X | X | X |  | X | X |  |  |  | X | X | 7 |
| 10 | Incontinence |  |  |  |  | X |  | X | X | X |  | X | X | 6 |
| 11 | Hypertension |  | X | X | X | X | X |  |  |  |  | X |  | 6 |
| 12 | Dizziness | X | X | X | X |  |  |  |  | X |  |  |  | 5 |
| 13 | Dysphagia |  |  | X |  |  |  | X |  |  | X | X | X | 5 |
| 14 | Heart Failure | X | X |  |  |  | X |  |  |  |  |  | X | 4 |
| 15 | Arthritis |  |  |  |  |  | X |  |  |  | X | X | X | 4 |
| 16 | Vision |  |  |  |  | X |  |  | X | X |  |  | X | 4 |
| 17 | Fracture |  |  |  |  |  | X |  | X | X |  |  |  | 3 |
| 18 | Pain |  |  |  |  |  | X |  |  | X |  |  | X | 3 |
| 19 | Feet |  |  | X |  |  |  |  | X | X |  |  |  | 3 |
| 20 | Anxiety |  |  |  |  | X |  |  |  | X |  |  |  | 2 |
| 21 | Skin Ulcer |  |  |  |  |  |  |  |  | X |  |  | X | 2 |
| 22 | Anorexia |  |  | X |  |  |  |  |  |  |  |  | X | 2 |
| 23 | Falls |  |  |  |  |  | X |  |  |  |  |  | X | 2 |
| 24 | Ckd |  |  |  |  |  | X |  |  |  |  |  |  | 1 |
| 25 | Dyspnea |  |  |  |  |  |  |  |  | X |  |  |  | 1 |
| 26 | Pvd |  |  | X |  |  |  |  |  |  |  |  |  | 1 |
| 27 | Thyroid |  |  |  |  | X |  |  |  |  |  |  |  | 1 |
| 28 | Af |  |  | X |  |  |  |  |  |  |  |  |  | 1 |
| 29 | Hypotension |  |  |  |  |  |  |  |  |  |  |  |  | 0 |
| 30 | Parkinsonism |  |  |  |  |  |  |  |  |  |  |  |  | 0 |
| 31 | Peptic |  |  |  |  |  |  |  |  |  |  |  |  | 0 |
| 32 | Heart Valve Disease |  |  |  |  |  |  |  |  |  |  |  |  | 0 |
| 33 | Sleep |  |  |  |  |  |  |  |  |  |  |  |  | 0 |

Decision Tree

|  | Algorithm | ACFI+Low Cost EFI | | | Low Cost EFI | | | None | | | GA Selected Subset | | | Usage |
| --- | --- | --- | --- | --- | --- | --- | --- | --- | --- | --- | --- | --- | --- | --- |
| 1 | Number of Features | 5 | 10 | 15 | 5 | 10 | 15 | 5 | 10 | 15 | 5 | 10 | 15 |  |
| 2 | Diabetes | X | X | X | X |  | X | X |  | X | X | X | X | 10 |
| 3 | Osteoporosis | X | X | X |  |  | X | X |  | X | X | X | X | 9 |
| 4 | Heart Failure | X | X | X | X | X |  |  |  |  | X | X | X | 8 |
| 5 | Dizziness |  |  |  | X | X | X |  | X | X |  | X |  | 6 |
| 6 | Dysphagia |  |  |  | X | X | X |  |  | X |  | X | X | 6 |
| 7 | Ihd |  | X | X |  |  | X |  | X | X |  |  | X | 6 |
| 8 | Hearing |  | X | X |  | X | X |  |  |  |  |  | X | 5 |
| 9 | Cva | X |  |  |  |  |  | X | X | X | X |  |  | 5 |
| 10 | Hypertension |  | X | X | X | X | X |  |  |  |  |  |  | 5 |
| 11 | Incontinence |  |  | X |  |  |  |  | X | X |  |  | X | 4 |
| 12 | Pvd |  | X |  |  |  |  | X |  | X |  |  |  | 3 |
| 13 | Pain |  | X | X |  |  | X |  |  |  |  |  |  | 3 |
| 14 | Memory Cognitive |  |  |  |  |  | X | X | X |  |  |  |  | 3 |
| 15 | Anorexia |  |  | X |  | X |  |  |  |  |  |  | X | 3 |
| 16 | Falls |  |  |  |  |  | X |  |  | X |  |  | X | 3 |
| 17 | Depression |  |  |  |  |  |  |  | X | X |  |  | X | 3 |
| 18 | Feet | X | X | X |  |  |  |  |  |  |  |  |  | 3 |
| 19 | Peptic |  |  |  |  |  |  |  |  | X |  |  | X | 2 |
| 20 | Thyroid |  |  |  |  |  |  |  |  | X |  | X |  | 2 |
| 21 | Sleep |  |  |  |  |  |  |  | X |  |  | X |  | 2 |
| 22 | Hypotension |  |  |  |  |  |  |  |  |  | X |  | X | 2 |
| 23 | Dyspnea |  |  |  |  |  | X |  | X |  |  |  |  | 2 |
| 24 | Copd |  |  |  |  |  |  |  | X |  |  | X |  | 2 |
| 25 | Anxiety |  |  |  |  | X | X |  |  |  |  |  |  | 2 |
| 26 | Fracture |  |  |  |  |  |  |  |  |  |  |  | X | 1 |
| 27 | Af |  |  |  |  |  |  |  |  | X |  |  |  | 1 |
| 28 | Ckd |  |  | X |  |  |  |  |  |  |  |  |  | 1 |
| 29 | Heart Valve Disease |  |  |  |  |  |  |  |  |  |  |  | X | 1 |
| 30 | Arthritis |  |  |  |  |  |  |  | X |  |  |  |  | 1 |
| 31 | Vision |  |  |  |  | X |  |  |  |  |  |  |  | 1 |
| 32 | Parkinsonism |  |  |  |  |  |  |  |  |  |  |  |  | 0 |
| 33 | Skin Ulcer |  |  |  |  |  |  |  |  |  |  |  |  | 0 |

Random Forest

|  | Algorithm | ACFI+Low Cost EFI | | | Low Cost EFI | | | None | | | GA Selected Subset | | | Usage |
| --- | --- | --- | --- | --- | --- | --- | --- | --- | --- | --- | --- | --- | --- | --- |
| 1 | Number of Features | 5 | 10 | 15 | 5 | 10 | 15 | 5 | 10 | 15 | 5 | 10 | 15 |  |
| 2 | Ihd | X | X | X | X |  | X |  | X | X | X | X | X | 10 |
| 3 | Cva |  |  | X |  | X | X | X | X | X | X | X | X | 9 |
| 4 | Memory Cognitive | X | X | X | X | X | X |  | X |  |  | X |  | 8 |
| 5 | Depression |  | X | X |  |  |  | X | X | X | X | X | X | 8 |
| 6 | Diabetes | X | X | X | X | X | X | X |  | X |  |  |  | 8 |
| 7 | Osteoporosis | X | X | X |  | X | X | X |  |  |  |  | X | 7 |
| 8 | Hearing |  | X | X |  | X | X |  |  | X |  | X | X | 7 |
| 9 | Copd |  |  | X |  | X | X |  | X | X | X |  |  | 6 |
| 10 | Hypertension | X | X | X | X | X | X |  |  |  |  |  |  | 6 |
| 11 | Incontinence |  |  | X |  |  |  |  | X | X | X |  | X | 5 |
| 12 | Heart Failure |  |  | X |  | X | X |  |  |  |  | X | X | 5 |
| 13 | Fracture |  |  |  |  |  | X |  |  | X |  | X | X | 4 |
| 14 | Dysphagia |  |  |  | X |  |  |  | X |  |  | X | X | 4 |
| 15 | Pvd |  |  | X |  |  | X |  | X |  |  |  | X | 4 |
| 16 | Falls |  |  | X |  |  | X |  |  |  |  | X | X | 4 |
| 17 | Dizziness |  | X | X |  |  |  |  | X |  |  |  |  | 3 |
| 18 | Feet |  |  |  |  | X | X |  |  |  |  |  |  | 2 |
| 19 | Ckd |  |  |  |  | X | X |  |  |  |  |  |  | 2 |
| 20 | Anxiety |  |  |  |  |  | X |  |  |  |  |  | X | 2 |
| 21 | Thyroid |  |  |  |  |  |  |  |  | X |  |  | X | 2 |
| 22 | Sleep |  |  | X |  |  |  |  |  |  |  |  |  | 1 |
| 23 | Pain |  |  |  |  |  |  |  |  |  |  |  | X | 1 |
| 24 | Peptic |  |  |  |  |  |  |  |  | X |  |  |  | 1 |
| 25 | Parkinsonism |  |  |  |  |  |  |  | X |  |  |  |  | 1 |
| 26 | Anorexia |  |  |  |  |  |  |  |  |  |  |  | X | 1 |
| 27 | Arthritis |  |  |  |  |  |  |  |  |  |  | X |  | 1 |
| 28 | Dyspnea |  |  |  |  |  |  |  |  | X |  |  |  | 1 |
| 29 | Af |  |  |  |  |  |  | X |  |  |  |  |  | 1 |
| 30 | Vision |  |  |  |  |  |  |  |  | X |  |  |  | 1 |
| 31 | Hypotension |  |  |  |  |  |  |  |  |  |  |  |  | 0 |
| 32 | Heart Valve Disease |  |  |  |  |  |  |  |  |  |  |  |  | 0 |
| 33 | Skin Ulcer |  |  |  |  |  |  |  |  |  |  |  |  | 0 |
