# Supplementary material for: Evolving Hybrid Partial Genetic Algorithm Classification Model for Cost-effective Frailty Screening: Investigative Study
Source: JMIR Aging. 2022 Oct 7;5(4):e38464. doi: 10.2196/38464 (PMC9587492; doi:10.2196/38464)
Supplement: Multimedia Appendix 3 [file aging_v5i4e38464_app3.docx]

Multimedia Appendix 3: Low-Cost Features Selected for Models Built With GA Selected Subset

Linear Regression

Complex Health Care Domain, Complex Health Care, Dressing and Undressing

Support Vector Machine

Complex Health Care Domain, Complex Health Care, Verbal Behaviour, Dressing and Undressing, Toilet Completion

Decision Tree

Activities of Daily Living, Wandering, Dressing and Undressing, Continence

Random Forest

Activities of Daily Living, Wandering, Toilet Completion, Continence
